# Icodextrin-associated generalized exfoliative skin rash in a CAPD patient: a case-report

**DOI:** 10.1186/s12882-018-1071-6

**Published:** 2018-10-25

**Authors:** Vassilios Liakopoulos, Panagiotis I Georgianos, Paraskevi Demirtzi, Vasilios Vaios, Theofanis Kalathas, Pantelis E Zebekakis

**Affiliations:** Peritoneal Dialysis Unit, 1st Department of Medicine, AHEPA Hospital, Aristotle University of Thessaloniki, St. Kyriakidi 1, GR54636, Thessaloniki, Greece

**Keywords:** Icodextrin, Hypersensitivity, Peritoneal dialysis, Skin rash

## Abstract

**Background:**

Icodextrin is a starch-derived, water soluble glucose polymer, which is used as an alternative to glucose in order to enhance dialytic fluid removal in peritoneal dialysis patients. Although the safety and efficacy of icodextrin is well-established, its use in everyday clinical practice has been associated with the appearance of skin rashes and other related skin reactions.

**Case presentation:**

Herein, we report the rare case of a 91-year-old woman with a history of severe congestive heart failure, who initiated continuous ambulatory peritoneal dialysis with icodextrin-based dialysate solutions and 15 days after the initial exposure to icodextrin developed a generalized maculopapular and exfoliative skin rash extending over the back, torso and extremities. Discontinuation of icodextrin and oral therapy with low-dose methyl-prednisolone with quick dose tapering improved the skin lesions within the following days.

**Conclusions:**

This case report highlights that skin hypersensitivity is a rare icodextrin-related adverse event that should be suspected in patients manifesting skin reactions typically within a few days or weeks after the initial exposure.

## Background

Icodextrin, a starch-derived iso-osmolar, high-molecular weight (16,200 Da) glucose polymer, is extensively used as the osmotic agent for the long dwell in patients receiving either continuous ambulatory peritoneal dialysis (CAPD) or continuous cycling peritoneal dialysis in order to enhance dialytic fluid removal [1, 2]. Despite the fact that the efficacy and safety of icodextrin is well-documented, the use of this agent has been associated with relatively high incidence of skin rash [2–5]. In most cases, icodextrin-related skin lesions are limited to the palms and soles and do not mandate the permanent withdrawal of icodextrin from the peritoneal dialysis regimen. In rare occasions, however, exposure to icodextrin may be accompanied by widespread exfoliative dermatitis, acute generalized exanthematous pustulosis, maculopapular rash or chronic lymphocytic vasculitis [6–11]. These severe episodes of skin hypersensitivity typically occur within a few days or weeks of the initial exposure and necessitate the permanent discontinuation of icodextrin [6–11].

In this article, we report the rare case of a generalized maculopapular and exfoliative skin rash approximately 2 weeks after the introduction of icodextrin in a 91 year old CAPD patient.

## Case presentation

We report the case of a 91-year-old woman, who developed a generalized maculopapular, exfoliative skin eruption extending to the back, torso and extremities 15 days after initiation of CAPD with the use of icodextrin dialysate solutions. The medical history of the patient included stage IV congestive heart failure according to the New York Heart Association (NYHA) classification secondary to massive tricuspid valve failure and severe mitral valve deficiency, chronic atrial fibrillation (cAF) and stage 4 chronic kidney disease (CKD) with an estimated-glomerular-filtration-rate (eGFR) of 20 ml/min/1.73m^2^. A Tenckhoff peritoneal catheter was surgically inserted and after a prolonged hospitalization in the Peritoneal Dialysis Unit due to leakage from the peritoneal catheter exit site, the patient was discharged and her CAPD regimen included 2 icodextrin exchanges per day (2 X 1.0 L icodextrin). The daily dose of icodextrin relative to the patient’s body weight was 41.7 ml/kg and the peritoneal ultrafiltration volume was 600 ml/day. The patient was re-evaluated 7 days later and the CAPD regimen was intensified with the addition of another exchange during the day with dialysate glucose 1.36% (2 X 1.0 L icodextrin and 1 X 1.0 L glucose 1.36%). Peritoneal ultrafiltration achieved with the intensified regimen was approximately 800 ml/day.

**Fig. 1:**
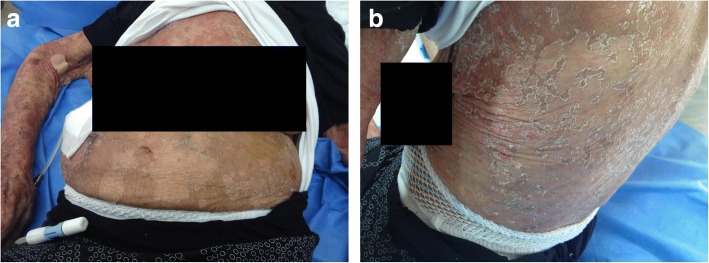
**a** Generalized exfoliative skin rash over the torso and upper extermities; **b** exfoliative skin rash in the back

On Day 15 after her initial exposure to icodextrin, the patient was admitted to the Peritoneal Dialysis Unit because of a widespread maculopapular and exfoliative skin rash of abrupt onset extending over her abdomen, arms, legs and back (Fig. 1a and b). Her physical examination on admission revealed a normal body temperature (36.7 °C), blood pressure 105/60 mmHg, pulse rate 70 bpm, oxygen saturation 95% in the room air and absence of abnormal clinical signs from the chest auscultation and palpation of the abdomen. The peritoneal effluent was macroscopically clear and the white blood cell (WBC) count in the fluid was 35 cells/mm^3^, indicating absence of peritonitis. As shown in Table 1, standard laboratory tests revealed a normal WBC count with absence of eosinophilia (WBC: 8630 cells/μL; Neutrophils: 84%; Lymphocytes: 7%; Eosinophils: 1.1%), stable renal function without significant electrolyte disturbances (serum urea: 141 mg/dl, serum creatinine: 2.19 mg/dl, serum potassium: 3.6 mEq/L, serum sodium: 135 mEq/L), whereas inflammatory biomarkers remained within the normal range (c-reactive-protein: 0.8 mg/dl, normal range: 0.1–0.8 mg/dl; erythrocyte sedimentation rate: 18 mm/hour). The levels of immunoglobulin IgE in the serum were also normal (20.8 IU/ml, normal range 10–100 IU/ml).

**Table 1:** Patient’s laboratory values on the day of admission

Parameter	Value
WBC (cells/μL)	8630
Neut/Lymph (%)	84/7
Mono/Eosin (%)	6/1.1
Hematocrit (%)	37.8
Hemoglobin (g/dL)	12.2
PLTs (cells/μL)	193,000
Serum glucose (mg/dl)	112
Serum urea (mg/dl)	141
Serum creatinine (mg/dl)	2.19
Serum potassium (mEq/L)	3.6
Serum sodium (mEq/L)	135
Serum calcium (mg/dL)	8.6
Serum phosphate (mg/dL)	3.2
AST/ALT (U/L)	14/9
CPK (U/L)	16
LDH (U/L)	119
CRP (mg/dL)	0.8
Erythrocyte sedimentation rate (mm/hour)	18
IgE (IU/mL)	20.8
INR/aPTT	1.05/30.9

*Abbreviations: WBC* white blood cells, *PLTs* platelets, *ALT* alanine aminotransferase, *AST* aspartate aminotransferase, *CPK* Creatine Phosphokinase, *LDH* lactate dehydrogenase, *CRP* c-reactive-protein

With respect to her medications, the patient was receiving oral therapy with digoxin (0.25 mg/d), furosemide (125 mg twice daily), eplerenone (25 mg/d), and folic acid (5 mg/d); the patient was also on darbepoetin alfa (40 μgr/week) subcutaneously for the treatment of CKD-related anemia as well as tinzaparin (3500 IU/d) as anti-coagulant therapy due to the history of cAF. The above regimen remained unchanged since her initial admittance to the hospital until the appearance of the skin rash. Notably, the medical history failed to uncover the use of any other drugs or substances that could be causally associated with the adverse skin reaction. Moreover, the patient reported no previous history of allergic reaction or known allergies.

The approximately 15-day-long period after the initial exposure to icodextrin along with the negative work-up for other drug-inducible allergic reactions set the suspicion of skin hypersensitivity to icodextrin. On this basis, we decided the discontinuation of icodextrin and modified the CAPD regimen using glucose 3.86% and glucose 1.36% dialysate solutions (2 X 1.5 L glucose 3.86% and 1 X 1.5 L glucose 1.36% per day). The replacement of icodextrin with hypertonic dialysate glucose solutions produced a similar peritoneal ultrafiltration volume of 800 ml/day. The patient was also initiated on oral therapy with methyl-prednisolone 32 mg daily with gradual tapering of the administered dose at weekly intervals. The clinical response was satisfactory and the skin rash improved within 7 days after icodextrin discontinuation. Unfortunately, 2 weeks later, the patient was admitted to our Department with clinical signs of fecal peritonitis that was attributed to colonic rupture and died after a major surgery in the Intensive Care Unit of our Hospital.

## Discussion and conclusions

This case report highlights the development of a generalized maculopapular and exfoliative skin rash as a rare, but serious complication associated with icodextrin use in patients undergoing peritoneal dialysis. The reported in observational and randomized controlled studies incidence of skin rash associated with icodextrin use is highly variable, ranging from 2.3% up to 18.9% [3, 5, 12]. In a 2013 meta-analysis of 3 randomized controlled trials (incorporating data from 755 patients), the risk of developing skin rash was not significantly higher among patients exposed to icodextrin in comparison with those exposed to glucose-containing dialysate solutions [Relative Risk (RR): 2.51; 95% Confidence Interval (CI): 0.59–10.72, *P* = 0.20] [3]; cessation of icodextrin as a result of incident skin rash was necessary in 4.3% of the participants [3]. This high variability in the reported incidence of adverse skin reactions is possibly reflective of the variability in the reporting criteria across studies, since the documentation of the etiologic association of icodextrin and/or the differentiation of icodextrin-related skin rash from other skin manifestations commonly occurring in uremic patients is not always clear.

Twice-daily administration of icodextrin is commonly used as a therapeutic approach to improve volume control in patients with ultrafiltration failure or as a glucose-sparing intervention in patients treated with hypertonic glucose-containing solutions. Observational studies have provided evidence that compared with the usual once-daily administration, “double-dose” of icodextrin has not been associated with higher incidence of skin rash or other adverse reactions [13, 14]. On this basis, we have reasons to believe that the occurrence of the exfoliative skin reaction in the case we describe cannot be explained by the administration of icodextrin in a twice-daily regimen.

Icodextrin-related skin hypersensitivity should be suspected in patients developing skin rash typically within a few days or a few weeks after initial exposure [6–11], in the absence of any other profound etiology for the allergic reaction (e.g. modification in the orally administered drugs and changes in the patient’s diet or lifestyle habits). Additional strength to the diagnosis of icodextrin-related skin hypersensitivity is provided by the improvement or full remission of the skin lesions within a few days or weeks of withdrawing icodextrin from the peritoneal dialysis regimen [6–11]. This is in line with pharmacokinetic properties of this agent, since icodextrin is shown to have a plasma half-life of 14.7 h and its metabolites are eliminated from plasma within 3 to 7 days after icodextrin discontinuation, depending on the residual renal function [15]. Skin tests with icodextrin-containing patches, pricks or intra-dermal tests cannot reliably confirm the diagnosis, since these re-challenging tests were unable to reproduce the skin rash in previously reported cases of acute exanthematous generalized pustulosis-like eruption that occurred shortly after exposure to icodextrin [4]. Notably, skin tests are reported to have a very low sensitivity and specificity in diagnosing such drug-induced skin reactions [4].

The patient of our case did not present peripheral eosinophilia and most importantly, the generalized skin rash was not accompanied by fever, lymphadenopathy, or involvement of other systems and organs. The presence of peripheral eosinophilia, although pathognomonic, is an inconsistent manifestation of drug-induced allergic eruptions [16]. Similarly to the patient of our case, icodextrin use has never previously been associated with the drug rash with eosinophilia and systemic symptoms (DRESS) syndrome [17], which is a rare but potentially life-threatening drug-induced hypersensitivity reaction, characterized by fever, rash, leucocytosis with eosinophilia and a variety of moderate-to-severe systemic manifestations with a prolonged latency period between the initial drug exposure and disease onset [17].

The exact allergenic icodextrin epitopes responsible for the adverse skin reactions are not yet identified. Icodextrin is partially absorbed from the peritoneum via the peritoneal lymphatic drainage. Icodextrin is metabolized via alpha amylase into maltose and other glucose polymers [18]. The chemical structure of icodextrin is very close to that of the naturally occurring dextran, which has been used as a plasma expander or anti-coagulant agent and has been associated with a number of allergic reactions, including anaphylaxis [19]. The main structural differentiation between icodextrin and dextran is the polymer linkages α-1,4 and α-1,6, respectively. The reported incidence of allergic reactions in patients treated with dextran-containing solutions is as high as 50% [20, 21]. In addition, the presence of impurities introduced into the icodextrin molecule during the manufacturing process (e.g., icodextrin batches with high peptidoglycan content) has been in the past associated with episodes of sterile peritonitis characterized by monocytosis in the peritoneal effluent [22]. Biopsy studies showed peritoneal monocyte infiltration and suggested a type IV hypersensitivity reaction mediated through dendritic cells [23]. Sensitization against icodextrin-related molecules, such as dextran, with formation of cross-reactive antibodies or progressive sensitization against icodextrin itself may be implicated in the complex pathophysiology of adverse skin reactions observed in patients exposed to this agent [4].

In conclusion, icodextrin is a useful osmotic agent with documented benefits, particularly in peritoneal dialysis patients with high transporter status and impaired dialytic fluid removal. However, clinicians should be aware of and suspect icodextrin-related skin hypersensitivity, mainly on the basis of the chronological association between the timing of the initial exposure to icodextrin and the onset of the skin rash.

## Abbreviations

cAF: Chronic atrial fibrillation
CAPD: Continuous ambulatory peritoneal dialysis
CI: Confidence interval
CKD: Chronic kidney disease
Dress: Drug rash with eosinophilia and systemic symptoms
eGFR: Estimated-glomerular-filtration-rate
NYHA: New York Heart Association
RR: Relative risk
WBC: White blood cell
